# Markers of inflammation predicts long-term mortality in patients with acute coronary syndrome – a cohort study

**DOI:** 10.1186/s12872-025-04608-9

**Published:** 2025-03-15

**Authors:** Jacob Odeberg, Anders Halling, Michael Ringborn, Michael Freitag, Marie Louise Persson, Ivar Vaara, Lennart Råstam, Håkan Odeberg, Ulf Lindblad

**Affiliations:** 1 Department of Protein Science, Science for Life Laboratory Stockholm, CBH, KTH Royal Institute of Technology, Stockholm, 100 44 Sweden; 2 Department of Clinical Medicine, Faculty of Health Science, Arctic University of Tromsö (UiT), Tromsö, N 9037 Norway; 3 Division of Internal Medicine, University Hospital North Norway (UNN), Tromsö, Norway; 4 Department of Hematology, Coagulation Unit, Karolinska University Hospital, Stockholm, Sweden; 5 Department of Medicine, Solna, Karolinska Institute, Stockholm, SE 171 77 Sweden; 6 Department of Clinical Sciences in Malmö, Lund University, Malmö, Sweden; 7 Thoracic Center, Blekinge County Hospital Karlskrona, Karlskrona, Sweden; 8 Department of Laboratory Medicine, Blekinge County Hospital Karlskrona, Karlskrona, Sweden; 9 School of Public Health and Community Medicine/Primary Health Care, University of Gothenburg, Göteborg, Sweden

**Keywords:** Inflammation, Acute coronary syndrome, Unstable angina, Myocardial infarction, Prognosis, Cardiac disease mortality

## Abstract

**Background:**

Chronic low-grade inflammation is a well-known risk factor for coronary heart disease (CHD) and future cardiovascular events. Anti-inflammatory therapy can reduce the risk of ischemic cardiovascular disease (CVD) events following myocardial infarction (MI). However, it remains unknown to what extent inflammation at the time of an acute event predicts long-term outcomes. We explored whether routine blood measurements of inflammatory markers during an acute coronary syndrome (ACS) are predictive of long-term mortality.

**Methods:**

In a cohort of 5292 consecutive patients admitted to a coronary intensive care unit with suspected ACS over a four-year period in the Carlscrona Heart Attack Prognosis Study (CHAPS), 908 patients aged 30–74 years (644 men, 264 women) were diagnosed with MI (527) or unstable angina (UA) (381). A 10-year follow-up study was conducted using Swedish national registries, with total mortality and cardiac mortality as primary outcomes.

**Results:**

Long-term total and cardiac mortality were significantly associated with higher leukocyte counts (e.g., neutrophils, monocytes, p ≤ 0.001), higher levels of inflammatory biomarkers (e.g., C-reactive protein, Serum Amyloid A, fibrinogen, p ≤ 0.001), and elevated neutrophil–lymphocyte ratio (NLR) (*p* < 0.001) and monocyte-lymphocyte ratio (MLR) (*p* = 0.002), all measured at ACS admission. These associations were independent of ACS diagnosis.

**Conclusion:**

Our results suggest that level of inflammation at ACS presentation—beyond its established role as a major CHD risk factor—also predicts long-term mortality following ACS. Notably, inflammation at the time of the event was a stronger predictor of long-term mortality than the acute event outcome itself. However, limitations include the observational study design, moderate sample size, and absence of modern high-sensitivity cardiac biomarkers and contemporary ACS management strategies in this cohort. The results should therefore be interpreted in the context of historical clinical practice. While our model-wise complete-case approach ensured consistency, missing data remains a potential source of bias. Future studies in larger, more contemporary cohorts are needed to validate these findings and refine risk stratification strategies.

**Supplementary Information:**

The online version contains supplementary material available at 10.1186/s12872-025-04608-9.

## Introduction

Atherosclerosis is a slowly progressing disease often marked by a long, asymptomatic subclinical phase that can culminate an acute coronary syndrome (ACS), manifested as either myocardial infarction (MI) or unstable angina (UA). ACS is usually a late complication, and often the first manifestation of the underlying atherosclerotic disease. The immediate trigger for an ACS event is typically a rupture (fissure) of a vulnerable atherosclerotic coronary artery plaque or a superficial endothelial erosion, both leading to thrombus formation [1]. These pathologies appear to be differently represented across ACS type [2–4]; ruptured plaques are predominant in ST-elevation myocardial infarction (STEMI), whereas endothelial erosions are more commonly observed in non-ST elevation myocardial infarction (NSTEMI) [4]. It is, however, not known whether such differences would affect long term mortality after an ACS event.

A chronic low-grade inflammatory process is considered to play a central pathogenic role in the development and progression of atherosclerosis, as well as in the acute thrombotic event in manifested as an ACS [5]. In a previous study based on the Carlscrona Heart Attack Prognosis Study (CHAPS), we demonstrated that blood cell profiles and the degree of inflammation at the time of admission were associated with the outcomes of ACS, differentiating between outcomes such as MI and UA [6]. Our data further suggested that a state of pre-existing low-grade inflammation could predispose individuals to different severities of ACS.

Since our initial research, there have been temporal shifts in the clinical presentation and underlying mechanisms of ACS in most Western countries. Hospitalization and death rates for STEMI have declined, with a relative increase in NSTEMI occurrences and changes in observed plaque characteristics underlying luminal occlusive thrombosis [4]. The rupturing lipid loaded culprit plaques, usually seen with STEMI, have become less dominant and plaques with erosion of the endothelium as initiator of thrombosis more frequent [4]. This can be ascribed to improvements in controlling traditional risk factors, especially dyslipidaemia. Even with excellent control of this risk factor, other risk elements persist, prompting a continued search for other anti-atherosclerotic therapies, recently reviewed by Peter Libby and Brenden Everett [7]. Recent studies have demonstrated the potential benefits of anti-inflammatory therapies in reducing ischemic events following myocardial infarction [8, 9]. In light of these findings, which highlight the protective effects of such therapies on post-infarction prognosis, we here aimed to explore whether individual differences in inflammatory markers in patients with ACS, as measured at time of the acute event, are associated with long-term prognosis. In a registry based 10-year follow up in CHAPS, we investigated if biomarkers of inflammation, measured at the time of hospital admission, were predictive of long-term mortality (cardiac disease mortality and total mortality).

## Materials and methods

### Study design

Based on CHAPS we performed an observational cohort sub-study including patients diagnosed with a MI or UA aged 30–74 years at admission.

### Patient recruitment

The CHAPS cohort has previously been described in detail [10]. In brief, 5292 consecutive patients admitted to the coronary care unit with acute chest pain (indicative of a possible ACS) at Blekinge Hospital, Karlskrona, between January 26 1992 and January 25 1996 were recruited. Patients who presented to the emergency room (ER) with recent or ongoing chest pain were at this time by routine directly transferred to the coronary care unit. Of these, 2992 were between 30–74 years. The diagnosis was set at discharge from the hospital by one of two experienced cardiologists. Based on this diagnosis, patients with MI or UA were identified. In patients admitted more than once during the inclusion period, only the first classifying admittance was included as an ‘event’ (UA or MI) in the analysis. Informed consent was obtained from all included patients before enrolment and the study complies with the Declaration of Helsinki.

### Outcome measures

Cardiac disease mortality and total mortality during the follow up period.

### Acute coronary syndrome patients

A diagnosis of ACS was confirmed in 908 of the eligible patients aged 30–74 years of age (644 men and 264 women) [10]. Two groups were identified: (i) patients experiencing at least one acute MI during the inclusion period [527] or (ii) patients experiencing no acute MI, but having at least one episode of UA during the inclusion period (381). In 900 of these patients complete data were available to perform longitudinal data analyses. The diagnostic criteria of acute MI were fulfilled if at least two of the following criteria were met: (i) A history of chest pain of at least 15 min duration, (ii) a rise of cardiac markers (CK-MB and ASAT) to at least twice the upper limit of normality, or (iii) characteristic ECG changes typical for MI e.g. change of ST segment and/or of T-waves and/or appearance of new Q-waves**.** These criteria included both patients with ST-elevation MI (STEMI) and non-ST elevation MI (NSTEMI). A diagnosis of UA was made when patients fulfilled all of the following criteria: (i) no evidence of MI, (ii) during the preceding 48 h acute chest pain of increased/modified character to any previously experienced, and (iii) angina pectoris diagnosed and medically treated before admission, or alternatively, angina pectoris ascertained by clinical evaluation, including a bicycle exercise test prior to discharge from the hospital [10]. Patients with post-infarction angina and secondary angina were not included. Patients admitted to the coronary care unit were initially treated with aspirin, and in case of ongoing chest pain, also nitrates and morphine. In patients meeting STEMI criteria, thrombolysis with streptokinase was administreted (193 of 527 patients with MI). If the diagnosis of MI was based on cardiac markers only, thrombolysis was not given. Acute coronary artery intervention was not available at this hospital at the time of the enrolment. Patients diagnosed with ACS were discharged with continuous aspirin treatment unless contraindications were present.

### Ethical approval

Carlscrona Heart Attack Prognosis Study (CHAPS) was approved by the Regional Ethical Review Board, Lund, Sweden, (EPN 2009/762 and LU 298–91). All patients gave their informed consent before taking part.

### Risk factors

Information on basic characteristics and risk factors were recorded from patient history at admission and/or extracted from earlier medical files, and the information was also verified at discharge from the hospital [10]. Smoking status was defined as current smoker or non-smoker. Patients who had quit smoking > 1 month prior to admission were classified as non-smokers. Hypertension was defined as a physician’s diagnosis prior to hospital admittance. In general, these patients were treated with blood pressure-lowering medications. Diabetes was defined as a previous diagnosis, and included diabetes treated with diet only, treated with oral medication only, or patients treated with insulin.

### Laboratory analyses

Blood samples for laboratory analyses were collected at hospital admission and processed at the certified hospital laboratory following standardized protocols. The inflammatory biomarkers included in this study were selected based on their routine availability in clinical practice as standardized assays in the hospital laboratory during the study. Hematological variables, including blood cell counts and indices, as well as plasma fibrinogen, were analyzed using routine diagnostic methods on fresh samples. Blood cell counts were measured in EDTA whole blood using the ADVIA 2120 system (Siemens, Germany), and plasma fibrinogen was assessed in sodium citrate samples on a Trombotrack instrument (Nycomed, Norway). Data were extracted from the hospital’s electronic laboratory records and entered into the study database. High-sensitivity C-reactive protein (hsCRP) and Serum Amyloid A protein (SAA) were analyzed in samples stored at −80 °C until analysis. After thawing, both biomarkers were measured on the BN ProSpec system (Siemens, Germany), with results directly recorded into the database. The laboratory maintains strict quality assurance, adhering to guidelines from the Association of Clinical Chemists in Sweden, with external control conducted via EQUALIS (Sweden) and Bio-Rad UKNEQAS (United Kingdom). Instruments from Roche and Siemens were validated per the IVD directive, and verification included intra-assay precision, detection limits, measurement accuracy, interferences, and pre-analytical factors. All laboratory results included in the study were within validated intra-assay ranges for each method.

### Mortality data

Information on date of death and causes of death was obtained from the Centre for Epidemiology at the Swedish National Board of Health and Welfare for a period from 28th January 1992 to 10th May 2017. All registered causes of death were obtained, but only the primary cause of death was used. Using ICD 9- or ICD 10 diagnoses of the primary cause of death a variable was made containing 4 categories to be used in competing-risks survival regression: 1. Cardiac (ICD 9: 402, 404, 410, 411, 412, 413, 414, 427, 428, ICD 10: I48, I50, I11, I13, I20, I21, I23, I24, I25), 2. Cerebrovascular (ICD 9: 430, 431, 432, 433, 434, 436, 437, ICD 10: I60, I61, I62, I63, I64, I65, I66, I67), 3. Venous tromboembolism (ICD 9: 415, 451, ICD 10: I26, I80, I81) and 4. Other reasons.

### Statistical methods

STATA version 16 (Stata Corporation, Texas, USA) was used for all data analyses. Standard methods were used for descriptive statistics. All-cause mortality was analysed for short- (0–28 days) and long-term mortality (29 days −10 years). The division of short- and long-term mortality was based on the AHA case definitions for acute coronary heart disease in epidemiology and clinical research studies [11]. When analysing short term all-cause mortality Cox regression was used. When analysing long term mortality both Cox regression was used for analysing all-cause mortality and competing-risks survival regression was used to study cardiac disease specific mortality over the same time period. Associations with specific biomarkers were analysed with diagnosis (UA/MI), age and sex in the model and presented by hazard ratio (HR) with 95% confidence intervals (CI) and p-values.

Age was entered into the regressions as a continuous variable. Plasma levels of hs-CRP were dichotomized at 2 mg/L to categorize individuals into high and low risk groups. This cut off is based on the JUPITER study, which selected individuals at high vascular risk because of an enhanced inflammatory response as indicated by hsCRP levels ≥ 2 mg/L [12]. For other biomarkers to categorise into risk groups, these were divided into tertiles, using tertile 1 as reference in the analyses. The neutrophil to lymphocyte ratio (NLR) and monocyte to lymphocyte ratio (MLR) was calculated by dividing neutrophil and monocyte cell counts (10^9^/L), respectively, with lymphocyte cell counts (10^9^/L). Tests for trend was performed by entering tertiles as a linear variable into the regression, and the results presented as p values.

To manage missing data, we employed a model-wise complete-case analysis approach, i.e. only participants with complete data for the variables included in that particular model were analyzed. In the different multivariate analyses performed, subjects with missing data for any included marker were automatically excluded, leaving about 70% left in the regression for the full models. To assess whether missing data introduced bias, we performed an additional analysis using Multiple Imputation by Chained Equations (MICE) for hsCRP and neutrophils. We conducted 20 and 50 imputations and compared hazard ratios (HRs) from the imputed models with those obtained from model-wise complete-case analysis, adjusting for age and sex. Since the differences in HR estimates were minimal across imputations, and due to uncertainty regarding the missing data mechanism (i.e., whether data were Missing Completely at Random [MCAR], Missing at Random [MAR] or Not Missing at Random [NMAR]), we retained model-wise complete-case analysis throughout the study.

### Patient and public involvement

Patients or the public were not involved in the design, or conduct, or reporting, or dissemination plans of our research.

## Results

The study population comprised 908 patients with ACS; 527 were diagnosed with myocardial infarction (MI) and 381 with unstable angina (UA) (see Tables 1, 2, and 3 for descriptive data). Due to lack of follow-up data, 8 patients were excluded. Within the first 28 days, there were 48 deaths (33 men, 15 women), of which 43 were due to cardiac disease. Of these, 42 occurred in the MI group, indicating that the cause of death was related to cardiac complications of the acute MI. Between 29 days and 10 years, there were a total of 306 deaths, 200 of which were due to cardiac causes (125 in MI, 75 in UA) and 106 due to other causes (65 in MI, 41 in UA). From 10 to 25 years, there were 144 cardiac deaths (79 in MI, 65 in UA) and 175 deaths due to other causes (88 MI, 87 UA).

**Table 1:** Clinical characteristics of patients at inclusion

	Men (*n* = 644)		Women (*n* = 264)	
	m	(SD)	m	(SD)
Age (years)	63.0	(8.6)	65.5	(8.0)
Serum cholesterol (mmol L^−1^)	6.0	(1.3)	6.6	(1.4)
hsCRP (mmol/L)	9.3	(21.9)	9.0	(21.4)
	n	(%)	n	(%)
Myocardial infarction (MI)	394	(61.1)	133	(50.4)
Unstable Angina (UA)	250	(38.9)	131	(49.6)
Hypertension	169	(27.2)	71	(28.1)
Diabetes	92	(14.8)	50	(19.7)
Smoking (current)	148	(24.1)	43	(17.1)

Data taken from Odeberg *et. al.* [10]. Hypertension and diabetes were defined as a physician's diagnosis prior to hospital admittance. Missing data serum cholesterol (*n* = 102), hypertension (*n *= 34), diabetes (*n *= 34), smoking (*n* = 43).

**Table 2:** Cause of death at follow up in relation to diagnosis at inclusion

	Men (*n* = 637)		Women (*n* = 263)	
	n	(%)	n	(%)
Deaths <29 days^a^	33	(5.2)	15	(5.7)
- Cardiac disease mortality	31	(4.9)	12	(4.6)
MI	30	(4.7)	12	(4.6)
UA	1	(0.2)	0	(0)
- Other mortality	2	(0.3)	3	(1.1)
MI	1	(0.2)	1	(0.4)
UA	1	(0.2)	2	(0.8)
Deaths 29 days −10 years^a^	221	(35.7)	85	(32.3)
- Cardiac disease mortality	121	(19.0)	44	(16.7)
MI	78	(12.2)	28	(10.6)
UA	43	(6.8)	16	(6.1)
- Other mortality	100	(15.7)	41	(15.6)
MI	63	(9.9)	21	(8.0)
UA	37	(5.8)	20	(7.6)

*MI *myocardial infarction, *UA *unstable angina. The diagnosis was set at point of discharge from hospital following acute coronary syndrome event

^a^Total mortality

**Table 3:** Categorized blood biomarkers of inflammation at inclusion

			Men (*n* = 644)	Women (*n* = 264)
		Range	n (%)	n (%)
hsCRP mg/L		≥2	341 (59.3)	141 (63.5)
Fibrino (g/L)	Tert 1	1.5–3.3	233 (40.5)	77 (33.8)
	Tert 2	3.4–4.0	159 (27.7)	73 (32.0)
	Tert 3	4.1–10.0	183 (31.8)	78 (34.2)
SAA (mg/L)	Tert 1	0.111–3.25	209 (36.3)	57 (25.7)
	Tert 2	3.26–7.44	191 (33.2)	75 (33.8)
	Tert 3	7.45–1570	175 (30.4)	90 (40.5)
Leuco (10^9^/L)	Tert 1	2.49–7.39	196 (32.6)	85 (35.4)
	Tert 2	7.4–9.8	198 (32.9)	84 (35.0)
	Tert 3	9.82–80.9	207 (34.4)	71 (29.6)
Neutro (10^9^/L)	Tert 1	0.14–4.79	191 (32.3)	84 (36.5)
	Tert 2	4.81–7.04	196 (33.1)	78 (33.9)
	Tert 3	7.05–20.06	205 (34.6)	68 (29.6)
Baso (10^9^/L)	Tert 1	0–0.039	229 (40.7)	95 (43.0)
	Tert 2	0.04–0.059	174 (30.9)	60 (27.1)
	Tert 3	0.06–0.33	160 (28.4)	66 (29.9)
Eosino (10^9^/L)	Tert 1	0–0.06	155 (27.2)	84 (36.8)
	Tert 2	0.07–0.14	186 (32.6)	77 (33.8
	Tert 3	0.15–9.12	229 (40.2)	67 (29.4)
Lympho (10^9^/L)	Tert 1	0.16–1.32	206 (34.8)	71 (30.9)
	Tert 2	1.33–1.88	191 (32.3)	80 (34.8)
	Tert 3	1.89–75.33	195 (32.9)	79 (34.3)
Mono (10^9^/L)	Tert 1	0.04–0.4	167 (28.4	116 (50.7)
	Tert 2	0.41–0.56	212 (36.0)	59 (25.8)
	Tert 3	0.57–1.60	210 (35.7)	54 (23.6)
T-cyt (10^9^/L)	Tert 1	85–198	228 (38.1)	54 (2.6)
	Tert 2	199–247	182 (30.4)	94 (39.3)
	Tert 3	248–680	188 (31.4)	91 (38.1)
T-mcv (fL)	Tert 1	6.5–8.8	221 (39.4)	69 (31.4)
	Tert 2	8.9–9.4	167 (29.8)	78 (35.5)
	Tert 3	9.5–46.0	173 (30.8)	73 (33.2)
NLR ()	Tert 1	0.075–2.73	187 (31.6)	84 (36.5)
	Tert 2	2.74-4.87	205 (34.7)	82 (35.7)
	Tert 3	4.87–48.3	199 (33.7)	64 (27.8)
MLR ()	Tert 1	0.216–0.892	166 (28.2)	111 (48.5)
	Tert 2	0.892–1.477	201 (34.2)	68 (29.7)
	Tert 3	1.481–7.142	221 (37.6)	50 (21.8)

Data are means (m) and standard deviations (SD), or numbers (n) and proportions (%).

Tert – tertile; hsCRP - high sensitivity CRP; Fibrino - fibrinogen; SAA - Serum Amyloid A; Leuco -total leukocyte cell count; Neutro-neutrophil cell count; Baso-Basophil cell count; Eosino-eosinophil cell count; Lympho - lymphocyte cell count; Mono - monocyte cell count; T-cyt -thrombocyte cell count; T-mcv - thrombocyte median cell volume; NLR - neturophile to lymphocyte ratio; MLR – monocyte to lymphocyte ratio. Plasma levels of hsCRP were dichotomized at 2 mg/L, while other biomarkers were divided in tertiles for categorical comparisons

Missing data hsCRP (*n* = 111), fibrinogen (*n* = 105), S-Amyloid A (*n* = 111), leukocytes (*n* = 67), neutrophils (*n* = 86), basophils (*n* = 124), eosinophils (*n* = 110), lymphocytes (*n* = 86), monocytes (*n* = 90), thrombocyte cell count (*n* = 71), T-mcv (*n* = 127), NLR (*n* = 87), MLR (*n* = 91). Data taken from Odeberg et. al. [6].

We first analyzed how ACS diagnosis (MI vs. UA) was associated with short-term (0–28 days) and long-term (29 days—10 years) mortality after an ACS (Table 4). A diagnosis of MI over UA was associated with both short-term (HR = 5.38 [1.84–15.74], *p* = 0.002) and long-term total mortality (HR = 1.27 [1.01–1.61], *p* = 0.041). When analyzed separately as cardiac disease mortality, a diagnosis of MI over UA was significantly associated with long-term cardiac disease mortality (HR 1.41 [1.02–1.94], *p* = 0.035). Short-term total mortality was primarily due to cardiac disease mortality (43 out of 48 deaths, Table 2). Sex was not associated with either short-term (HR 0.63 [0.27–1.42]) or long-term (HR 1.24 [0.96–1.60]) total mortality. Age was associated with long-term (HR 1.11 [1.06–1.17], *p* < 0.001) but not short-term (HR 1.91 [0.84–5.15]) total mortality. When analyzing mortality beyond 10 years to the end of the follow-up period (25 years), we found no significant difference between MI and UA with respect to cardiac disease mortality (HR 1.225 [0.975–1.54]) or total mortality (HR 1.10 [0.94–1.28]).

**Table 4:** Short term (<29 days) and long term (29 days – 10 years) follow up mortality in relation to acute coronary syndrome diagnosis

	HR	95% CI	*p*
0–28 days			
Total mortality (MI vs UA)	5.38	1.84–15.74	0.002
29 days – 10 yrs			
Total mortality (MI vs UA)	1.27	1.01–1.61	0.041
Cardiac mortality (MI vs UA)	1.41	1.02–1.94	0.035

Associations between diagnosis at inclusion and mortality following an ACS were estimated using cox regression and expressed as hazard ratios (HR) with 95% confidence intervals (95% CI), adjusted for differences in sex and age.

We next analyzed whether inflammatory variables measured in samples taken at admission for an ACS were associated with mortality at follow-up. Statistically significant associations with short-term total mortality was found for a number of blood markers of inflammation when analyzed as categorized variables, adjusting for sex and age along with the ACS outcome of the recent acute event (Table 5). For serum amyloid A (SAA), leukocytes, neutrophils, and basophils, significant associations were found with the third tertile, using tertile 1 as the reference, and the trend for association was also significant (*p* < 0.05) for these (Table 5). Furthermore, there was a non-significant trend for association with a higher neutrophil to lymphocyte ratio (NLR) but not monocyte to lymphocyte ratio (MLR) (Table 5). Long-term total mortality, adjusted for differences in age and sex, was significantly associated with hsCRP ≥ 2 mg/L (HR 1.82 [1.39–2.38] *p* < 0.001) and higher tertiles of fibrinogen, SAA, total leukocytes, neutrophils, monocytes and NLR (p for trend ≤ 0.001), as well as with higher tertiles of MLR (*p* = 0.002) and basophiles (*p* = 0.007) (Table 6). To assess the potential impact of missing data on these analyses, we performed multiple imputation for hsCRP and neutrophils using 20 and 50 imputations. The hazard ratios (HRs) obtained from the imputed datasets showed only minor variations compared to the model-wise complete-case analysis. For hsCRP, the HR for the second level was 1.83 [1.42–2.37] with 20 imputations, and 1.84 [1.42–2.38] with 50 imputations, and for neutrophils, the HRs for the second and third tertiles remained nearly unchanged with 20 imputations (1.35 [1.01–1.82], 1.93 [1.45–2.56]) and 50 imputations (1.35 [1.00–1.82], 1.91 [1.43–2.56]), compared to 1.37 [1.01–1.85] and 1.95 [1.46–2.62] in the model-wise complete-case analysis (Table 6). Since these variations were minimal, we decided to retain model-wise complete-case analysis throughout the study to ensure transparency and consistency while avoiding potential biases introduced by imputation assumptions.

**Table 5:** Short term (<29 days) total mortality in relation to categorized inflammatory markers at acute coronary syndrome event (adjusted for age sex and ACS outcome)

		***HR***	***95% CI***	
hsCRP > 2 mg/L		1.66	0.46–5.94	n.s.
Fibrino	Tert 1	1.00		*p *for trend 0.403
	Tert 2	4.23	1.08–16.63	
	Tert 3	1.90	0.49–7.32	
SAA	Tert 1	1.0		*p* for trend 0.045
	Tert 2	7.99	0.75–84.85	
	Tert 3	8.95	1.03–77.69	
Leuco	Tert 1	1.00		*p* for trend 0.019
	Tert 2	3.02	0.49–7.32	
	Tert 3	6.70	1.19–37.60	
Neutro	Tert 1	1.00		*p* for trend 0.009
	Tert 2	3.78	0.66–21.54	
	Tert 3	9.00	1.69–47.83	
Eosino	Tert 1	1.00		p for trend 0.038
	Tert 2	1.21	0.79–1.83	
	Tert 3	1.54	1.02–2.33	
Baso	Tert 1	1.00		*p* for trend 0.105
	Tert 2	5.44	1.19–24.89	
	Tert 3	4.68	1.24–17.62	
Lymfocyt	Tert 1	1.00		*p* for trend 0.149
	Tert 2	2.59	0.76–8.84	
	Tert 3	2.14	0.75–6.16	
Monocyt	Tert 1	1.00		*p *for trend 0.344
	Tert 2	1.08	0.26–4.42	
	Tert 3	1.71	0.52–5.68	
T-cyt	Tert 1	1.00		*p* for trend 0.115
	Tert 2	1.66	0.48–5.72	
	Tert 3	2.77	0.77–9.94	
T-mcv	Tert 1	1.00		*p* for trend 0.972
	Tert 2	0.26	0.06–1.19	
	Tert 3	1.28	0.37–4.41	
NLR	Tert 1	1.00		*p* for trend 0.17
	Tert 2	21.5	2.14–215.34	
	Tert 3	7.97	0.92–69.03	
MLR	Tert 1	1.00		*p* for trend 0.80
	Tert 2	1.96	0.56–6.96	
	Tert 3	0.96	0.28–3.32	

Associations between inflammatory markers and short term mortality following an ACS were estimated using binary logistic regression and expressed as hazard ratios (HR) with 95% confidence intervals (95% CI), adjusted for differences in sex, age and diagnosis at inclusion

*Tert* tertile, *hsCRP* high sensitivity CRP, *Fibrino* fibrinogen, *SAA* Serum Amyloid A, Leuco-total leukocyte cell count; Neutro-neutrophil cell count; Eosino-eosinophil cell count; Baso-Basophil cell count; Lympho-lymphocyte cell count; Mono-monocyte cell count; T-cyt thrombocyte cell count; T-mcv thrombocyte median cell volume; NLR - neutrophile to lymphocyte ratio; MLR – monocyte to lymphocyte ratio. Plasma levels of hsCRP were dichotomized at 2 mg/L, while other biomarkers were divided in tertiles for categorical comparisons using tertile 1 as reference. The tertiles were then entered into the regression as a linear variable to test for trend

**Table 6:** Long term (29 days - 10 years) total mortality in relation to categorized inflammatory markers measured at acute coronary syndrome event (adjusted for age and sex)

		***HR***	***95% CI***	
hsCRP > 2 mg/L		1.82	1.39–2.38	*p <0.001*
Fibrino	Tert 1	1.00		*p for trend <0.001*
	Tert 2	1.29	0.93–1.79	
	Tert 3	2.61	1.86–3.66	
SAA	Tert 1	1.0		*p for trend <0.001*
	Tert 2	1.56	1.12–2.15	
	Tert 3	2.35	1.71–3.21	
Leuco	Tert 1	1.00		*p for trend <0.001*
	Tert 2	1.37	1.02–1.85	
	Tert 3	2.15	1.61–2.89	
Neutro	Tert 1	1.00		*p for trend 0.001*
	Tert 2	1.37	1.01–1.85	
	Tert 3	1.95	1.46–2.62	
Baso	Tert 1	1.00		*p for trend 0.007*
	Tert 2	1.61	1.20–2.15	
	Tert 3	1.69	1.26–2.27	
Eosino	Tert 1	1.00		*p for trend 0.366*
	Tert 2	0.94	0.7–1.27	
	Tert 3	1.15	0.95–1.53	
Lymfocyt	Tert 1	1.00		*p for trend 0.91*
	Tert 2	1.01	0.76–1.34	
	Tert 3	0.98	0.73–1.32	
Monocyt	Tert 1	1.00		*p for trend <0.001*
	Tert 2	1.29	0.95–1.75	
	Tert 3	1.75	1.30–2.36	
T-cyt	Tert 1	1.00		*p for trend 0.038*
	Tert 2	0.98	0.73–1.32	
	Tert 3	1.34	1.02—1.78	
T-mcv	Tert 1	1.00		*p for trend 0.078*
	Tert 2	0.73	0.54–0.97	
	Tert 3	0.78	0.58–1.05	
NLR	Tert 1	1.00		*p for trend 0.001*
	Tert 2	1.35	0.99–1.82	
	Tert 3	1.64	1.21–2.22	
MLR	Tert 1	1.00		*p for trend 0.002*
	Tert 2	1.19	0.87–1.63	
	Tert 3	1.59	1.18–2.14	

Associations between inflammatory markers and long term mortality following an ACS were estimated using cox regression and expressed as hazard ratios (HR) with 95% confidence intervals (95% CI), adjusted for differences in sex and age.

*Tert *tertile, *hsCRP *high sensitivity CRP, *Fibrino *fibrinogen, *SAA *Serum Amyloid A, Leuco-total leukocyte cell count; Neutro-neutrophil cell count; Eosino-eosinophil cell count; Baso-Basophil cell count; Lympho-lymphocyte cell count; Mono-monocyte cell count; T-cyt - thrombocyte cell count; T-mcv - thrombocyte median cell volume; NLR - neturophil to lymphocyte ratio; MLR – monocyte to lymphocyte ratio. Plasma levels of hsCRP were dichotomized at 2 mg/L, while other biomarkers were divided in tertiles for categorical comparisons using tertile 1 as reference. The tertiles were then entered into the regression as a linear variable to test for trend

When analyzed separately with respect to cardiac disease mortality, strong significant associations were found between long term mortality and higher levels of plasma biomarkers of inflammation (hsCRP, SAA, fibrinogen, p for trend ≤ 0.001) and with total leukocytes, neutrophils, monocytes, basophiles (p for trend ≤ 0.003 for all) (Table 7). Significant associations were also found with cell biomarker indices NLR (*p* = 0.016) and MLR (*p* = 0.004) (Table 7).

**Table 7:** Long term (29 days – 10 years) Cardiac disease mortality in relation to categorised inflammatory markers measured at the acute coronary syndrome event (adjusted for age and sex)

**Risk factors**		**HR**	**95% CI**	
hsCRP > 2 mg/L		1.87	1.30–2.70	
Fibrino	Tert 1	1.00		p for trend < 0.001
	Tert 2	1.26	0.80–1.98	
	Tert 3	2.38	1.58–3.57	
S-amyloid	Tert 1	1.0		p for trend 0.001
	Tert 2	1.54	0.99–2.40	
	Tert 3	2.08	1.36–3.19	
Leuco	Tert 1	1.00		p for trend < 0.001
	Tert 2	1.18	0.78–1.80	
	Tert 3	2.31	1.56–3.32	
Neutro	Tert 1	1.00		p for trend < 0.001
	Tert 2	1.28	0.84–1.97	
	Tert 3	2.28	1.54–3.40	
Eosino	Tert 1	1.00		p for trend 0.038
	Tert 2	1.21	0.79–1.83	
	Tert 3	1.54	1.02–2.33	
Baso	Tert 1	1.00		p for trend 0.003
	Tert 2	1.24	0.82–1.87	
	Tert 3	1.79	1.22–2.61	
Lymfocyt	Tert 1	1.00		p for trend 0.674
	Tert 2	0.92	0.62–1.35	
	Tert 3	1.10	0.75–1.63	
Monocyt	Tert 1	1.00		p for trend < 0.001
	Tert 2	1.52	0.98–2.36	
	Tert 3	2.49	1.64–3.78	
T-cyt	Tert 1	1.00		p for trend 0.92
	Tert 2	0.90	0.61–1.33	
	Tert 3	1.02	0.69–1.51	
T-mcv	Tert 1	1.00		p for trend 0.545
	Tert 2	0.87	0.58–1.29	
	Tert 3	0.89	0.59–1.34	
NLR	Tert 1	1.00		p for trend 0.016
	Tert 2	1.15	0.76–1.76	
	Tert 3	1.61	1.09–2.40	
MLR	Tert 1	1.00		p for trend 0.004
	Tert 2	1.04	0.66–1.63	
	Tert 3	1.77	1.19–2.64	

Associations between risk factors and long term (29 days – 10 years) mortality following an ACS were estimated using binary logistic regression and expressed as hazard ratios (HR) with 95% confidence intervals (95% CI), adjusting for differences in sex and age

Tert – tertile; hsCRP—high sensitivity CRP; Fibrino-fibrinogen; Leuco-total leukocyte cell count; Neutro-neutrophil cell count; Eosino-eosinophil cell count; Baso-Basophil cell count; Lympho-lymphocyte cell count; Mono-monocyte cell count; T-cyt thrombocyte cell count; T-mcv thrombocyte median cell volume. Plasma levels of hsCRP were dichotomized at 2 mg/L, while other biomarkers were divided in tertiles for categorical comparisons using tertile 1 as reference. The tertiles were then entered into the regression as a linear variable to test for trend

Adjusting also for the outcome of the original acute event, these associations remained highly significant for both total and cardiac disease mortality (Supplementary Tables S1 and S2, respectively (see Additional File 1)). Alternatively, stratifying by ACS outcome did not significantly alter the associations, as shown for hsCRP and neutrophils (see Additional file, Supplementary Table S5). Finally, to distinguish an inflammatory response to myocardial tissue necrosis from a possible pre-existing inflammation, we analyzed the levels of the inflammatory variables in relation to the duration from the onset of chest pain until blood sampling, as previously described [6]. Adjusting also for the duration of symptoms before sampling (> 240 min) did not influence our findings for either total mortality (Supplementary Table S3 (see Additional File 1)) or cardiovascular mortality (Supplementary Table S4 (see Additional File 1)).

## Discussion

### Principal findings

In this study, with a 10-year follow-up using registry data, we found that plasma biomarkers of inflammation (hsCRP, SAA, fibrinogen) measured at the acute event were associated with long-term mortality, independent of the original ACS diagnosis. Additionally, blood cell profiles (e.g., neutrophils, monocytes) at the acute event predicted both short- and long-term mortality. We also found that recently defined inflammatory composite biomarkers (e.g., NLR, MLR) were associated with long-term mortality. Our results suggest that standard blood biomarkers of inflammation measured at the acute event have prognostic value for future risk.

### Results in relation to previous studies

Chronic inflammation is a well-known driver of atherosclerosis progression and risk of future acute CVD events [5]. Previously, based on the CHAPS study, we showed that prothrombotic gene variants predisposed individuals to MI over UA as outcomes of ACS in a fibrinogen concentration-dependent manner [13]. A more pronounced state of inflammation at admission conferred an increased risk towards MI rather than UA [6]. We observed significant differences in blood cell profiles at the time of the event between patients diagnosed with MI and UA, suggesting important differences in the underlying pathology of ACS between these two groups [6].Our current findings that blood cell profiles and levels of plasma biomarkers of inflammation measured at admission are also associated with individual long-term prognosis (cardiac disease mortality and total mortality), independent of the original ACS classification (UA or MI), suggest that the inflammatory activity reflected by these markers, existing prior to the acute event [6], persists throughout the follow-up period. This supports the potential of targeting inflammation post-ACS as an intervention point. This is consistent with the findings of the Jupiter study [12], which demonstrated that in apparently healthy individuals without hyperlipidemia but with elevated baseline hsCRP levels, the statin rosuvastatin significantly reduced the incidence of major cardiovascular events, coinciding with a reduction in hsCRP levels [12]. For individuals in the placebo group with elevated baseline hsCRP levels, these levels remained stable throughout the observation period [14]. Those findings suggested a residual risk associated with inflammation beyond cholesterol-related risks. In the Canakinumab Anti-inflammatory Thrombosis Outcome Study (CANTOS), the inflammatory hypothesis of atherothrombosis was tested by blocking the IL-1 inflammatory signaling pathway with the human monoclonal anti-IL-1 antibody canakinumab [9]. The results showed that treatment reduced the risk of recurrent atherosclerotic events after a myocardial infarction [9], and that this reduction was proportional to the reduction in inflammatory biomarkers e.g. hsCRP [15]. In a sub-study of CANTOS, it was demonstrated that the incidence rates of atherosclerotic events and all-cause mortality decreased in parallel with a reduction in IL-6 levels [16]. This indicates the IL-1-to-IL-6-to CRP signalling pathway to constitute a major target for vascular protection in secondary prevention, which has been elaborated in recent reviews [17, 18]. The mechanisms are likely to be multifaceted, including the stimulating effect of IL-6 on bone marrow production of neutrophils. In a recent randomized, double-blind, placebo-control study (the COLCOT study), colchicine, which has been used as a remedy for the inflammation in gout since centuries, was shown to reduce the percentage of ischemic cardiovascular events among patient with a recent myocardial infarction [8]. It was also shown that earlier initiation of therapy < 3 days significantly reduced cardiovascular death and/or recurrent event compared to initiation within 4–7 days [19]. The results in COLCOT have been particularly attributed to the inhibiting effect on tubulin polymerization and microtubule formation in phagocytes like neutrophils and macrophages [20]. Together with our findings of a strong significant association between long term CHD mortality and neutrophil counts, this suggests a direct role of neutrophils in future CVD risk. Our results are also in line with findings in the STABILITY (Stabilization of Atherosclerotic plaque by initiation of Darapladib Therapy) trial [21]. In this long-term prospective study on patients with optimally treated stable CAD, white blood cell counts carried independent prognostic information on the risk of future cardiovascular events [21].

Overall, we find for both short and long term mortality a trend for increased risk with higher concentration of inflammatory markers or blood cell counts, where the highest risk is observed for the third tertile, with the exception of fibrinogen, where the second tertile is associated with short term mortality, but not the third. This is consistent with our previous observation in CHAPS that common structural variants (polymorphisms) in the platelet fibrinogen receptor Gp2b/3a and coagulation factor XIII were associated with MI over UA in the second tertile, but not the third when analysed with an effect model based on functional in vitro studies [13].

### Methodological aspects—strengths and limitations

A strength of the current study is the near complete follow up mortality data for the ACS cohort (900 out of 908 patients). Due to strict registration procedures at a national level based on the unique Swedish personal identification number, this registry is considered complete. Furthermore, the study was based in one centre with the same two cardiologists assessing and categorising all patients, using consistent criteria.

The patients were recruited before the introduction of PCI, CABG, and modern antithrombotic drugs in the standard management of ACS. These interventions would otherwise influence the outcome and diagnosis in the acute event. The absence of them at that time made it possible for us to identify progression to MI or UA as distinct outcome groups within the cohort. The results from our previous work based on the CHAPS cohort, where we have studied genetic and clinical risk factors and routine plasma biomarkers in relation to ACS outcome [6, 10, 13], support that the classification of ACS patients in CHAPS into these two groups represent distinct outcome entities. By using these outcomes as proxies for underlying pathology, we were able to explore hypotheses that differences in underlying pathological mechanisms in MI and UA affect long term mortality.

Importantly, although we find the outcome of ACS to MI over UA to be significantly associated with long-term mortality, mainly explained by cardiac disease mortality, the associations of inflammatory variables with long term mortality are independent of ACS outcome. This suggests that low grade inflammation at time of event is associated with long term mortality independent of possible differences in underlying pathology of UA and MI.

Prognostic models such as the GRACE (Global Registry of Acute Coronary Events) score, TIMI (Thrombolysis in Myocardial Infarction) score, and SEX-SHOCK score are widely used to assess short-term outcomes, particularly in-hospital or 30-day mortality following ACS [22–24]. These models incorporate acute phase clinical parameters such as heart rate, blood pressure, and cardiac biomarkers, which are highly relevant for immediate risk stratification in the acute setting however, their predictive accuracy declines over longer time frames [25]. Given our focus on 10-year mortality**,** acute clinical variables (e.g., hemodynamic instability, initial troponin levels, renal function at presentation) are less relevant than persistent inflammatory activity**,** which reflects long-term cardiovascular risk [16]. As our dataset lacks several key variables needed to compute these scores comprehensively, it is not possible to adjust and assess their impact on hazard ratios or associations in our results. However, by adjusting for ACS type (MI vs. UA)—which captures key prognostic elements such as ST-segment deviation and cardiac biomarkers—we indirectly account for short-term predictors. As we show, this did not significantly alter hazard ratios for long-term mortality (Supplementary Table S1-S4 (see Additional File 1)). Future studies could explore how inflammatory markers at presentation might complement or refine existing risk stratification models to improve long-term risk prediction.

There are limitations of the study that should be acknowledged. Biochemical analyses of fibrinogen and blood cells were performed over a period of four years, however the hospital routine laboratory used standardised and certified methods, providing consistency over time. Analyses of hsCRP and SAA were performed using frozen samples stored at −80 °C for 15 years; quality assurance work at the laboratory has shown that storage of samples at −80 oC did not influence determined hsCRP and SAA levels, and high sensitivity C-reactive protein (hsCRP) remains highly stable in long-term archived human serum [26]. Outcomes are strong and consistent with the age and sex adjusted model leaving 90% left in the regression. Furthermore, the overall patterns show a high internal consistency.

Our study demonstrates significant associations between several inflammatory biomarkers and long-term mortality, particularly in the highest tertile categories. For key markers such as fibrinogen, serum amyloid A (SAA), and leukocyte counts, highly significant results were observed in tertile 3, whereas tertile 2 did not consistently reach statistical significance. This pattern may reflect limited power to detect moderate effect sizes in intermediate tertiles, particularly for biomarkers with smaller effect sizes or less pronounced biological gradients. Nonetheless, the highly significant trends across tertiles provide robust evidence of association, reinforcing the relevance of these biomarkers to long-term mortality.

These findings suggest that the study has adequate statistical power to detect substantial differences in mortality risk, particularly for the most strongly associated biomarkers. However, the observational design and modest sample size for certain comparisons may limit the ability to detect weaker associations or more nuanced trends. Future studies in larger cohorts will be necessary to validate these findings, refine the observed trends in intermediate tertiles, and further explore associations for biomarkers with smaller effect sizes.

The choice of a tertile-based analysis was motivated by its simplicity, clinical relevance, and robustness, particularly in studies with moderate sample sizes like here, enabling straightforward interpretation [27]. This aligns with previous studies exploring biomarker-mortality associations, using categorized or dichotomized variables for ease of interpretation and comparison (e.g. [12]). More advanced methods like restricted cubic splines (RCS) could provide additional granularity in analyzing these types of data, especially in larger studies [28]. RCS are valuable for modeling non-linear relationships and exploring dose–response curves. However, their added complexity can obscure clinical relevance and require larger datasets for reliable estimation, particularly in sparse data regions. In a moderate-sized cohort like in our study, RCS introduces a risk of overfitting and can reduce statistical robustness. Additionally, the subjective selection of knots in RCS models can influence results and complicate interpretation [29]. Future research with larger cohorts and greater statistical power that allow for more detailed modeling could benefit from RCS, building on our findings to explore the shape of dose–response relationships and refine risk predictions.

A key methodological consideration in observational studies is how to handle missing data. In this study, we applied a model-wise complete-case analysis, where each analysis included only participants with complete data for the variables in that specific model. This differs from a dataset-wise complete-case analysis, where individuals missing data on any variable across all analyses would be excluded entirely, which in our study would significantly reduce the available sample size. We choose the model-wise approach to optimizes statistical power while maintaining internal consistency within each model. Sensitivity analyses showed consistent patterns in the available data, supporting the robustness of our findings. Our approach aligns with recommendations for handling missing data when the underlying mechanism cannot be reliably established [30]. However, to assess the potential impact of missing data, we conducted Multiple Imputation by Chained Equations (MICE) for hsCRP, which had the highest proportion of missing values, and for neutrophils, which had a proportion of missing values comparable to other significant cell index-based findings (e.g., NLR, MLR, monocytes).The imputed results showed only minimal changes in hazard ratios compared to the model-wise complete-case analysis. The validity of imputation methods such as MICE depends on assumptions about the missing data mechanism [31]. Given the uncertainty regarding whether the data whether data were Missing Completely at Random [MCAR], Missing at Random [MAR] or Not Missing at Random [NMAR]) and since the observed differences were negligible, we opted to retain the model-wise complete-case analysis approach throughout the study.

The definition of myocardial infarction (MI) used in this study was based on the diagnostic criteria available at the time of CHAPS cohort enrollment (1992–1996). Since then, the European Society of Cardiology (ESC) guidelines, particularly the Universal Definition of MI, have evolved significantly [32]. The increased detection of NSTEMI compared to unstable angina (UA) observed today can be partly attributed to the incorporation of high-sensitivity myocardial damage markers, such as troponin, into the current MI definition. These markers are far more sensitive than older biomarkers like CK/CK-MB [4]. Consequently, it is likely that some UA cases in our study would now be diagnosed as NSTEMI under modern criteria [32].

Importantly, the identified associations between inflammatory biomarkers and long-term mortality remained highly significant when adjusted for or stratified by the diagnosis set at the time. Thus, reclassification using modern criteria would not be expected to significantly alter the main conclusions of our study. Furthermore, while modern treatment strategies have significantly reduced coronary artery disease mortality [33], it is noteworthy that for more than half of patients suffering an acute event, this is the first indication of underlying atherosclerotic disease. These patients are naïve to CAD preventive treatment, making them comparable to the CHAPS cohort. However, as CHAPS is a single-center study, and risk factor profiles and treatment protocols, including targeted anti-inflammatory therapies, have evolved since it was conducted, the results may not be fully generalizable to a broader, modern population.

## Conclusions and possible clinical implications

In conclusion, while chronic low-grade inflammation is well established as a major risk factor for development of CHD and risk of future events, our study indicates that the level of inflammation present at time of acute event carries consequences for long term prognosis. Available routine biochemical markers that offer prognostic information in samples taken already at presentation of an ACS, independent of underlying pathology and acute diagnosis (UA or MI) offers an opportunity for early risk stratification, which could be of clinical value in terms of more advanced secondary prevention approach in addition to what is in clinical practice of today. The association of inflammatory blood cell profiles with long term prognosis raise several mechanistic hypotheses that warrant further investigation, such as the role of neutrophils in predisposing to future CVD events. Establishing such mechanisms at the cellular level could lead to optimisation of pharmacological treatment for patients that have suffered an acute event.

## Supplementary Information

Additional file 1. Supplementary table S1. Long term total mortality in relation to categorized inflammatory markers measured at acute coronary syndrome event. Supplementary table S2. Long term cardiac disease mortality in relation to categorised inflammatory markers measured at the acute coronary syndrome event. Supplementary table S3. Long term total mortality in relation to categorized inflammatory markers measured at acute coronary syndrome event. Supplementary table S4. Long term cardiac disease mortality in relation to categorized inflammatory markers measured at acute coronary syndrome event. Supplementary table S5. Associations between long term all-cause mortality, hsCRP and neutrophils in relation to ACS outcome, or adjusted for differences in sex, age and ACS outcome.

## Data Availability

The data used in this study include registry-based information, as well as laboratory data collected both during routine clinical management and through subsequent research activities conducted by the study team. Due to the sensitive nature of these data and to protect participant confidentiality, they cannot be made publicly available. However, de-identified data may be available from the authors upon reasonable request, contingent upon approval by the relevant ethical review boards and compliance with institutional and regulatory guidelines. Requests for data access should include a detailed proposal and will be evaluated on a case-by-case basis to ensure adherence to ethical standards and privacy regulations.

## Abbreviations

ACS: Acute Coronary Syndrome
MI: Myocardial Infarction
UA: Unstable Angina
CHD: Coronary Heart Disease
PCI: Percutaneous Coronary Intervention
CABG: Coronary Arterial Bypass Graft Surgery
hsCRP: High sensitivity C-Reactive Protein
SAA: Serum Amyloid A
OR: Odds Ratio
HR: Hazards Ratio
CI: Confidence Interval
