# Supplementary material for: Markers of inflammation predicts long-term mortality in patients with acute coronary syndrome – a cohort study
Source: BMC Cardiovasc Disord. 2025 Mar 15;25:190. doi: 10.1186/s12872-025-04608-9 (PMC11909928; doi:10.1186/s12872-025-04608-9)
Supplement: Supplementary file 1 — Additional file 1. Supplementary table S1. Long term total mortality in relation to categorized inflammatory markers measured at acute coronary syndrome event. Supplementary table S2. Long term cardiac disease mortality in relation to categorised inflammatory markers measured at the acute coronary syndrome event. Supplementary table S3. Long term total mortality in relation to categorized inflammatory markers measured at acute coronary syndrome event. Supplementary table S4. Long term cardiac disease mortality in relation to categorized inflammatory markers measured at acute coronary syndrome event. Supplementary table S5. Associations between long term all-cause mortality, hsCRP and neutrophils in relation to ACS outcome, or adjusted for differences in sex, age and ACS outcome. [file 12872_2025_4608_MOESM1_ESM.pdf]

Supplementary table S1. Long term (29 days - 10 years) total mortality in relation to categorized inflammatory markers measured at acute coronary syndrome event (adjusted for differences in sex and age and ACS diagnosis).

		HR	95% CI	
hsCRP >2 mg/L		1.77	1.35-2.31	*p <0.001*
Fibrino	Tert 1	1.00		*p for trend <0.001*
	Tert 2	1.28	0.92-1.78	
	Tert 3	2.41	1.79-3.23	
SAA	Tert 1	1.0		*p for trend <0.001*
	Tert 2	1.53	1.11-2.11	
	Tert 3	2.22	1.62-3.04	
Leuco	Tert 1	1.00		*p for trend <0.001*
	Tert 2	1.36	1.00-1.84	
	Tert 3	2.11	1.53-2.91	
Neutro	Tert 1	1.00		*p for trend <0.001*
	Tert 2	1.34	0.99-1.83	
	Tert 3	1.88	1.37-2.59	
Baso	Tert 1	1.00		*p for trend 0.001*
	Tert 2	1.62	1.21-2.17	
	Tert 3	1.65	1.22-2.22	
Eosino	Tert 1	1.00		*p for trend 0.242*
	Tert 2	0.97	0.72-1.31	
	Tert 3	1.19	0.89-1.60	
Lympho	Tert 1	1.00		*p for trend 0.951*
	Tert 2	1.03	0.78-1.37	
	Tert 3	0.99	0.73-1.33	
Monocyt	Tert 1	1.00		*p for trend* 0.001
	Tert 2	1.27	0.94-1.73	
	Tert 3	1.67	1.23-2.26	
T-cyt	Tert 1	1.00		*p for trend 0.057*
	Tert 2	0.97	0.72-1.31	
	Tert 3	1.31	0.99-1.74	
T-mcv	Tert 1	1.00		*p for trend* 0.135
	Tert 2	0.76	0.56-1.02	
	Tert 3	0.81	0.60-1.09	
NLR	Tert 1	1.00		*p for trend* 0.007
	Tert 2	1.32	0.97-1.79	
	Tert 3	1.55	1.12-2.11	
MLR	Tert 1	1.00		*p for trend* 0.007
	Tert 2	1.15	0.84-1.58	
	Tert 3	1.51	1.11-2.04	

Associations between inflammatory markers and long term mortality following an ACS were estimated using cox regression and expressed as hazard ratios (HR) with 95% confidence intervals (95% CI), adjusted for differences in sex, age and ACS diagnosis. hsCRP - high sensitivity CRP; Fibrino-fibrinogen; SAA - Serum Amyloid A; Leuco-total leukocyte cell count;

Neutro-neutrophil cell count; Eosino-eosinophil cell count; Baso-Basophil cell count; Lympho-lymphocyte cell count; Mono-monocyte cell count; T-cyt thrombocyte cell count; T-mcv thrombocyte median cell volume. Plasma levels of hsCRP were dichotomized at 2 mg/L, while other biomarkers were divided in tertiles for categorical comparisons using tertile 1 as reference. The tertiles were then entered into the regression as a linear variable to test for trend.

Supplementary table S2. Long term (29 days – 10 years) Cardiac disease mortality in relation to categorised inflammatory markers measured at the acute coronary syndrome event (adjusted for differences in sex and age and ACS diagnosis)

Risk factors		HR	95% CI	
hsCRP >2 mg/L		1.78	1.22-2.60	*p <0.001*
Fibrino	Tert 1	1.00		*p for trend <0.001*
	Tert 2	1.24	0.79-1.96	
	Tert 3	2.32	1.54-3.49	
S-amyloid	Tert 1	1.0		*p for trend 0.002*
	Tert 2	1.63	1.06-2.52	
	Tert 3	1.97	1.27-3.06	
Leuco	Tert 1	1.00		*p for trend <0.001*
	Tert 2	1.16	0.76-1.77	
	Tert 3	2.21	1.42-3.45	
Neutro	Tert 1	1.00		*p for trend <0.001*
	Tert 2	1.25	0.80-1.94	
	Tert 3	2.17	1.39-3.39	
Eosino	Tert 1	1.00		*p for trend 0.019*
	Tert 2	1.24	0.82-1.89	
	Tert 3	1.65	1.09-2.50	
Baso	Tert 1	1.00		*p for trend 0.004*
	Tert 2	1.26	0.83-1.89	
	Tert 3	1.75	1.20-2.57	
Lymfocyt	Tert 1	1.00		*p for trend 0.632*
	Tert 2	0.95	0.64-1.41	
	Tert 3	1.11	0.75-1.65	
Monocyt	Tert 1	1.00		*p for trend <0.001*
	Tert 2	1.50	0.96-2.33	
	Tert 3	2.37	1.55-3.62	
T-cyt	Tert 1	1.00		*p for trend 0.930*
	Tert 2	0.88	0.59-1.30	
	Tert 3	0.98	0.67-1.45	
T-mcv	Tert 1	1.00		*p for trend* 0.667
	Tert 2	0.91	0.61-1.36	
	Tert 3	0.92	0.61-1.38	
NLR	Tert 1	1.00		*p for trend* 0.061
	Tert 2	1.12	0.74-1.71	
	Tert 3	1.48	0.98-2.25	
MLR	Tert 1	1.00		*p for trend* 0.01
	Tert 2	1.00	0.64-1.57	
	Tert 3	1.67	1.11-2.51	

Associations between risk factors and long term (29 days – 10 years) mortality following an ACS were estimated using competing risk regression and expressed as hazard ratios (HR) with 95% confidence intervals (95% CI), adjusting for differences in sex and age and ACS

diagnosis. hsCRP - high sensitivity CRP; Fibrino-fibrinogen; Leuco-total leukocyte cell count; Neutro-neutrophil cell count; Eosino-eosinophil cell count; Baso-Basophil cell count; Lympho-lymphocyte cell count; Mono-monocyte cell count; T-cyt thrombocyte cell count; T-mcv thrombocyte median cell volume; NLR - neutrophile to lymphocyte ratio; MLR – monocyte to lymphocyte ratio. Plasma levels of hsCRP were dichotomized at 2 mg/L, while other biomarkers were divided in tertiles for categorical comparisons using tertile 1 as reference. The tertiles were then entered into the regression as a linear variable to test for trend.

Supplementary table S3. Long term (29 days - 10 years) total mortality in relation to categorized inflammatory markers measured at acute coronary syndrome event (adjusted for duration of symptoms, ACS diagnosis, age and sex)

		HR	95% CI	
hsCRP >2 mg/L		2.00	1.47-2.72	*p <0.001*
Fibrino	Tert 1	1.00		*p for trend <0.001*
	Tert 2	1.48	1,03-2.12	
	Tert 3	2.61	1.86-3.66	
SAA	Tert 1	1.0		*p for trend <0.001*
	Tert 2	1.55	1.08-2.22	
	Tert 3	2.47	1.73-3.52	
Leuco	Tert 1	1.00		*p for trend <0.001*
	Tert 2	1.31	0.94-1.84	
	Tert 3	1.92	1.34-2.75	
Neutro	Tert 1	1.00		*p for trend 0.001*
	Tert 2	1.21	1.25-2.57	
	Tert 3	1.74	1.25-2.57	
Baso	Tert 1	1.00		*p for trend 0.007*
	Tert 2	1.76	1.27-2.44	
	Tert 3	1.53	1.08-2.17	
Eosino	Tert 1	1.00		*p for trend 0.223*
	Tert 2	0.91	0.64-1.29	
	Tert 3	1.23	0.88-1.70	
Lympho	Tert 1	1.00		*p for trend 0.609*
	Tert 2	0.96	0.70-1.32	
	Tert 3	0.92	0.65-1.29	
Monocyt	Tert 1	1.00		*p for trend* 0.004
	Tert 2	1.34	0.95-1.90	
	Tert 3	1.68	1.18-2.39	
T-cyt	Tert 1	1.00		*p for trend 0.123*
	Tert 2	0.82	0.56-1.15	
	Tert 3	1.27	0.93-1.74	
T-mcv	Tert 1	1.00		*p for trend* 0.029
	Tert 2	0.74	0.53-1.04	
	Tert 3	0.69	0..49-0.98	
NLR	Tert 1	1.00		*p for trend* 0.011
	Tert 2	1.31	0.92-1.87	
	Tert 3	1.58	1.11-2.24	
MLR	Tert 1	1.00		*p for trend* 0.005
	Tert 2	1.17	0.81-1.69	
	Tert 3	1.62	1.14-2.30	

Associations between inflammatory markers and long term mortality following an ACS were estimated using binary logistic regression and expressed as hazard ratios (HR) with 95%

confidence intervals (95% CI), adjusted for differences in sex, age, ACS diagnosis and duration > 240 minutes since onset of symptoms. hsCRP - high sensitivity CRP; Fibrino-fibrinogen; SAA - Serum Amyloid A; Leuco-total leukocyte cell count; Neutro-neutrophil cell count; Eosino-eosinophil cell count; Baso-Basophil cell count; Lympho-lymphocyte cell count; Mono-monocyte cell count; T-cyt thrombocyte cell count; T-mcv thrombocyte median cell volume. Plasma levels of hsCRP were dichotomized at 2 mg/L, while other biomarkers were divided in tertiles for categorical comparisons using tertile 1 as reference. The tertiles were then entered into the regression as a linear variable to test for trend.

Supplementary table S4. Long term (29 days – 10 years) Cardiac disease mortality in relation to categorised inflammatory markers measured at the acute coronary syndrome event (adjusted for duration, diagnos and age and sex)

**Risk factors**		**HR**	**95% CI**	
hsCRP >2 mg/L		2.04	1.32-3.13	*p <0.001*
Fibrino	Tert 1	1.00		*p for trend <0.001*
	Tert 2	1.36	0.82-2.23	
	Tert 3	2.60	1.63-4.13	
S-amyloid	Tert 1	1.0		*p for trend <0.001*
	Tert 2	1.58	0.97-2.60	
	Tert 3	2.52	1.54-4.11	
Leuco	Tert 1	1.00		*p for trend 0.006*
	Tert 2	1.16	0.73-1.86	
	Tert 3	2.06	1..25-3.40	
Neutro	Tert 1	1.00		*p for trend 0.001*
	Tert 2	1.17	0.71-1.91	
	Tert 3	3.21	1.37-3.71	
Eosino	Tert 1	1.00		*p for trend 0.019*
	Tert 2	1.28	0.79-2.06	
	Tert 3	1.66	1.03-2.67	
Baso	Tert 1	1.00		*p for trend 0.022*
	Tert 2	1.47	0.94-2.31	
	Tert 3	1.66	1.05-2.60	
Lymfocyt	Tert 1	1.00		*p for trend 0.986*
	Tert 2	0.84	0.54-1.31	
	Tert 3	1.02	0.65-1.60	
Monocyt	Tert 1	1.00		*p for trend <0.001*
	Tert 2	1.75	1.06-2.90	
	Tert 3	2.72	1.68-4.41	
T-cyt	Tert 1	1.00		*p for trend 0.828*
	Tert 2	0.77	0.49-1.22	
	Tert 3	1.05	0.68-1.62	
T-mcv	Tert 1	1.00		*p for trend* 0.312
	Tert 2	0.89	0.57-1.38	
	Tert 3	0.77	0.47-1.28	
NLR	Tert 1	1.00		*p for trend* 0.053
	Tert 2	1.16	0.72-1.88	
	Tert 3	1.57	0.99-2.50	
MLR	Tert 1	1.00		*p for trend* 0.001
	Tert 2	1.09	0.64-1.84	
	Tert 3	2.11	1.34-3.34	

Associations between risk factors and long term (29 days – 10 years) mortality following an ACS were estimated using binary logistic regression and expressed as hazard ratios (HR) with

95% confidence intervals (95% CI), adjusting for differences in sex and age, diagnosis at inclusion, and duration > 240 minutes since onset of symptoms.

hsCRP - high sensitivity CRP; Fibrino-fibrinogen; Leuco-total leukocyte cell count; Neutro-neutrophil cell count; Eosino-eosinophil cell count; Baso-Basophil cell count; Lympho-lymphocyte cell count; Mono-monocyte cell count; T-cyt thrombocyte cell count; T-mcv thrombocyte median cell volume. Plasma levels of hsCRP were dichotomized at 2 mg/L, while other biomarkers were divided in tertiles for categorical comparisons using tertile 1 as reference. The tertiles were then entered into the regression as a linear variable to test for trend.

Supplementary table S5. Associations between long term (29 days - 10 years) all-cause mortality, hsCRP and neutrophiles in relation to ACS outcome

A:

**Risk factors (MI only)**		**HR**	**95% CI**	
hsCRP >2 mg/L		1.53	1.09-2.16	*p =0.015*
Neutro	Tert 1	1.00		*p for trend =0.005*
	Tert 2	1.37	0.88-2.12	
	Tert 3	1.78	1.17-2.71	

B:

**Risk factors (UA only)**		**HR**	**95% CI**	
hsCRP >2 mg/L		2.16	1.41-3.32	*p <0.001*
Neutro	Tert 1	1.00		*p for trend = 0.004*
	Tert 2	1.28	0.82-2.01	
	Tert 3	2.31	1.34-3.94	

C:

**Risk factors (All)**		**HR**	**95% CI**	
hsCRP >2 mg/L		1.77	1.35-2.31	*p <0.001*
Neutro	Tert 1	1.00		*p for trend <0.001*
	Tert 2	1.34	0.99-1.83	
	Tert 3	1.88	1.37-2.59	

D:

Risk factors (All)		***HR***	***95% CI***	
hsCRP >2 mg/L		1.82	1.39-2.38	*p <0.001*
Neutro	Tert 1	1.00		*p for trend 0.001*
	Tert 2	1.37	1.01-1.85	
	Tert 3	1.95	1.46-2.62	

Associations between long term all-cause mortality and hsCRP, or neutrophiles, were estimated using cox regression and expressed as hazard ratios (HR) with 95% confidence intervals (95% CI), adjusted for differences in sex, age and stratified by ACS diagnosis of MI (A) or UA (B). For comparison is shown associations with long term cardiac mortality for the whole group when adjusting for ACS outcome, age and sex (C) (data from supplementary table S1), or only for age and sex (D)(data from Table 3b). hsCRP - high sensitivity CRP; Neutro-neutrophil cell count; Plasma levels of hsCRP were dichotomized at 2 mg/L, while neutrophile counts were divided in tertiles for categorical comparisons using tertile 1 as reference. The tertiles were then entered into the regression as a linear variable to test for trend.
